# Minimal Aortic Injury Detected on Computed Tomography Angiography during Initial Trauma Imaging: Single Academic Level 1 Trauma Center Experience

**DOI:** 10.1055/s-0042-1757793

**Published:** 2022-12-20

**Authors:** Leila Rezai Gharai, Christopher Ovanez, William C. Goodman, Xiaoyan Deng, Dipankar Bandyopadhyay, Michel B. Aboutanos, Mark S. Parker

**Affiliations:** 1 Department of Diagnostic Radiology, Virginia Commonwealth University Health Systems, Richmond, Virginia; 2 Department of Radiology and Radiological Sciences, Johns Hopkins University Medical Institution, Baltimore, Maryland; 3 Radiology Associates of Richmond, Richmond, Virginia; 4 Department of Biostatistics, Virginia Commonwealth University, Richmond, Virginia; 5 Department of Surgery, Division of Acute Care Surgical Services, Virginia Commonwealth University Health Systems, Richmond, Virginia

**Keywords:** minimal aortic injury, CT angiography, blunt thoracic aortic injuries

## Abstract

**Background** Minimal aortic injury (MAI), a subtype of acute traumatic aortic injury, is being increasingly recognized with better imaging techniques. Given conservative management, the role of follow-up imaging albeit important yet has to be defined.

**Methods** All trauma chest computed tomography angiographies (CTAs) at our center between January 2012 and January 2019 were retrospectively reviewed for presence of MAI. MAIs were generally reimaged at 24 to 72 hours and then at a 7- and 30-day interval. Follow-up CTAs were reviewed for stability, progression, or resolution of MAI, along with assessment of injury severity scores (ISS) and concomitant injuries, respectively.

**Results** A total of 17,569 chest CTAs were performed over this period. Incidence of MAI on the initial chest CTA was 113 (0.65%), with 105 patients receiving follow-up CTAs. The first, second, third, and fourth follow-up CTAs were performed at a median of 2, 10, 28, and 261 days, respectively. Forty five (42.9%), 22 (21%), 5 (4.8%), and 1 (1%) of the MAIs were resolved by first, second, third, and fourth follow-up CTAs. Altogether, 21 patients showed stability (mean ISS of 16.6), and 11 demonstrated improvement (mean ISS 25.8) of MAIs. Eight patients had no follow-up CTA (mean ISS 21). No progression to higher-grade injury was observed. Advancing age decreased the odds of MAI resolution on follow-up. A possible trend ( *p* -value 0.22) between increasing ISS and time to resolution of MAIs was noted.

**Conclusion** In our series of acute traumatic MAIs diagnosed on CTA imaging, there was no progression of injuries with conservative management, questioning the necessity of sequential follow-up imaging.

## Introduction

**Fig. 1:**
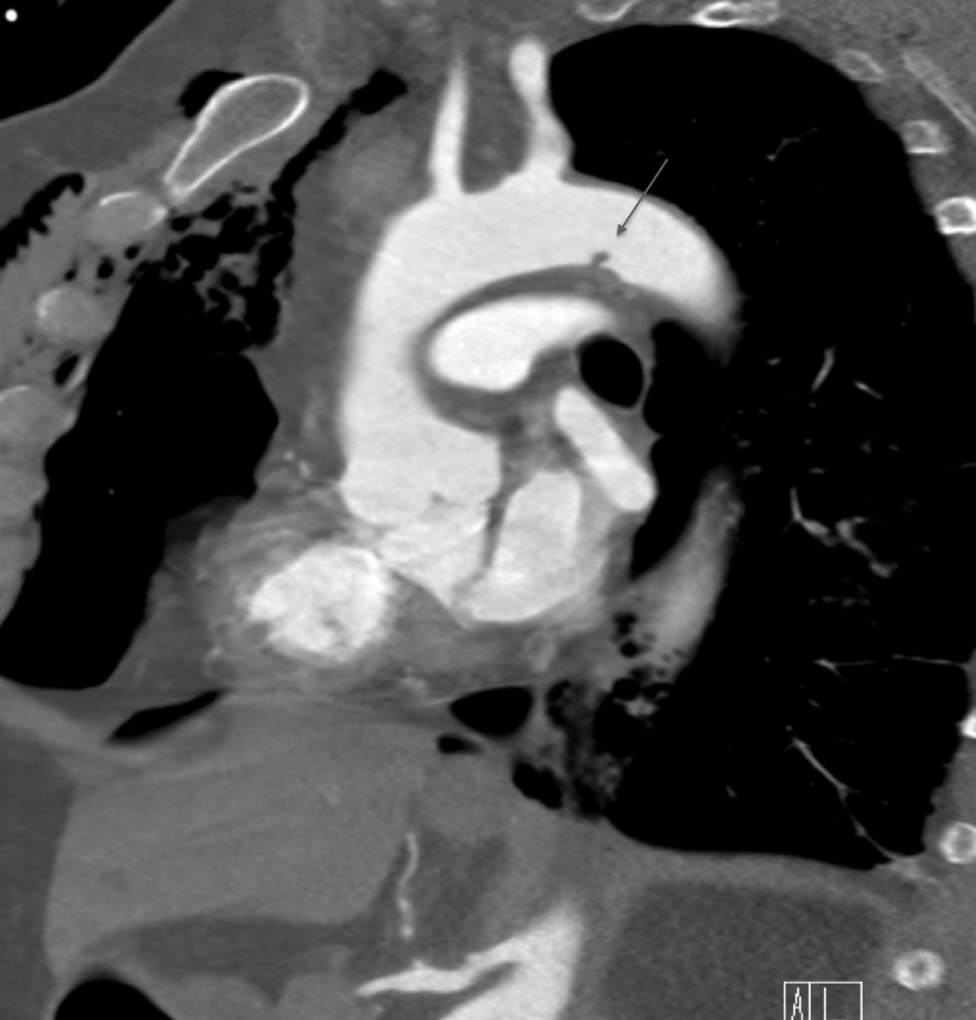
Contrast-enhanced sagittal oblique computed tomography angiography. (mediastinal windows) shows a <1.0 cm intimal flap, grade I injury, along the inferior aspect of the aortic arch (gray arrow). Note the hemopneumomediastinum and anterior chest wall subcutaneous air. This injury demonstrated complete healing on follow-up CTA imaging in 7-days' time.

Minimal aortic injury (MAI) is defined as a subtype of acute traumatic aortic injury (ATAI) in which the injury is limited to the aortic intima, manifesting as a subcentimeter round, triangular, or linear mural filling defect. This represents either a small intimal flap or focal thrombus, categorized as a grade I injury by the Society for Vascular Surgery 1 ( Fig. 1 ). MAI has been reported to be responsible for 10 to 28% of all ATAI injuries in studies by Gunn et al 2 and Malhotra et al. 3

Historically, single view portable trauma bay chest radiography was initially used as the screening imaging modality of choice to assess for indirect signs of potential aortic injury, usually manifesting as mediastinal hematoma. Suspected injury cases were then further imaged with catheter-based multiplanar aortography, which for decades was recognized as the “gold standard” for the diagnosis of ATAI. 4 Although chest radiography shows relatively high sensitivity for mediastinal hematoma (92%), the specificity is low (10%). Thus, this imaging modality alone cannot reliably identify the presence, significance, and/or severity of aortic injury. 5 In the late 1990s and early 2000s, the introduction of helical CT and its utilization as the initial screening test for suspected ATAI, dramatically reduced the clinical necessity of diagnostic transcatheter aortography. 6 Subsequent studies showed the absence of direct CT signs of aortic injury, such as intraluminal filling defect on helical CT, despite the presence of indirect signs of mediastinal hematoma, could obviate transcatheter aortography. 7 8 9 10 Some of the same studies proved CT to be more cost-effective in the diagnosis of ATAI than aortography. 8 9 Eventually, with the development of multidetector CT technology, CTA became the 21 ^st^ century “gold standard” for the diagnosis of ATAI, replacing invasive transcatheter aortography, with superior sensitivity and specificity. 8 9 11 12 13 Current 6th and 7th generation helical and multidetector computerized tomographies (MDCTs) provide exquisite spatial and temporal resolution not previously seen, surpassing that of preceding CT generations. Consequently, more subtle forms of ATAI are now being encountered.

The purpose of our study was to retrospectively review the MAI cases at our level 1 trauma center and evaluate the sequential evolution of thoracic aorta complications, if any, during the hospital course and afterward. We also sought to review the potential correlation between injury severity score (ISS) and MAI in our patient population to determine if a higher ISS portends a worse outcome.

## Materials and Methods

We received institutional review board (IRB) approval for this retrospective study. All data were encrypted to safeguard patients' confidentially in compliance with HIPAA regulations and individual patient consents were not required. mPower clinical management database software (Nuance Communications, Burlington, MA), integrated within our institution's McKesson (McKesson Corp., Irving, TX) Picture Archiving and Communication System (PACS), was utilized for our study.

At our institution, polytrauma patients admitted to the emergency department (ED) undergo multiorgan CTA based on a standardized evaluation by trauma surgeons.

The initial search of our institution's PACS database included all ED trauma chest CTAs performed as part of the initial trauma imaging work-up of blunt trauma victims presenting between January 2012 and January 2019. Our keyword search was then further refined to include the word “aortic” within this subset of trauma chest CTAs. The purpose was to broaden the search results within our specific time period and not limit cases based upon specific verbiage within dictated reports (i.e., injury, defect, abnormality, discontinuity, etc.). This enabled us to determine those patients with documented evidence of MAI.

Baseline trauma-survey chest CTAs are interpreted and/or over-read by either ED staff radiologists (with 8–35 years of experience) or Cardiothoracic Imaging Division staff radiologists (with 1–26 years of experience).

Upon identification of MAI on baseline or initial CTA imaging, follow-up CTA imaging over the next 24 to 72 hours is routinely recommended for reevaluation. Subsequently, and if indicated based on the initial follow-up CTA, repeat CTA imaging may then be recommended at 7 days and again at 30 days post injury to monitor for imaging signs of aortic injury progression, stability, or resolution. The final decision in management and follow-up imaging of the aortic injuries was determined by the consulting Cardiovascular–Thoracic Surgical Service.

We reviewed both the finalized radiology reports and imaging studies of all chest CTAs performed during the specified study time period. We also documented individual trauma victims' age, gender, mechanism of injury, total injuries incurred during the event, and identified evolution of MAI (i.e., stable, improved, resolved, or progressed), and the time of resolution of MAI (if applicable). Additionally, for the subset of MAI trauma patients, we searched the PACS system for follow-up chest CTAs and then retrospectively reviewed those images and reports for the sequential imaging findings. If follow-up CTA imaging was available, the natural history of the initial MAI, including stability, resolution, or progression was documented( Supplementary Fig. S1 ). We also reviewed electronic medical records—Cerner (Cerner Corp., MO)—of this subset of MAI trauma patients to document whether the patient survived, and if not, whether the cause of death was potentially related to an untreated traumatic aortic injury.

ISS is a standardized scoring system to assess an individual patient's critical state. There is a combined calculated score for six body regions (head and neck, face, chest, abdomen, extremities, and external). An increasing ISS denotes increasing severity of bodily injury. 14 ISS for each of the surviving patients was obtained from the trauma registry.

All baseline trauma chest CTAs were performed on either one of two ED CT scanners (Siemens SOMATOM Definition Edge, Forchheim, Germany; Siemens SOMATOM Definition AS + , Forchheim, Germany). Injectable iso-osmolar contrast media (e.g., Iohexol 350, Iopamidol 370 or Iodixanol 320) was administered at the rate of 4 mL/s. CTA imaging was acquired at 120 kVp and a pitch factor of 2.6, reconstructed in axial 2.0 mm × 1.0 mm slices. Multiplanar reconstructions were created in soft tissue and lung windows (sharp CT reconstruction kernel). All follow-up CTAs were obtained on an inpatient CT scanner (Siemens SOMATOM Definition Flash, Siemens Healthineers, Forchheim, Germany).

### Statistical Methods

All necessary data were extracted from the electronic medical records, our institution's McKesson PACS, and mPower database software. Logistic regression analysis was used to analyze the relationship between outcome, final follow-up CTA results, and the ISS. Other explanatory factors (age, gender) were also examined in the model. All analyses were performed with SAS (Statistical Analysis System, Cary, NC) software (v.9.4). The two-sided significance level was set to 5% for assessing the significance of the estimated parameters.

## Results

Between January 2012 and January 2019, 20,749 adult (age >18 years) blunt trauma encounters were registered at our institution. Out of a total of 17,569 blunt trauma chest CTAs performed over the study period, 113 MAIs were identified on the initial chest CTA (0.65% incidence), with 105 MAI patients receiving follow-up CTA imaging. The overall number of patients with blunt traumatic aortic injuries (BTAIs) of various grades were 118. Eight trauma victims received no follow-up CTA imaging, whether discharged or hospitalized (three due to other non-survivable injuries, four based on the treating surgeons' decision, and one based on the patient's personal choice). In total, 71 (67.6%) of the 105 MAI survivors were males and 34 females. The average male age was 42.2 years (range: 18–92 years), and the average female age was 52.4 years (range: 17–90 years). Including both genders, the average age for patients sustaining an MAI was 45.5 years ( Table 1 ). The most common mechanism of injury documented within Emergency Medical Services reports and ED notes for 99% of our subset of MAI trauma victims was motor vehicle collision, either involving a stationary object such as a tree or traffic pole or another vehicle. ISS was obtained by accessing the data in the Trauma Registry.

**Table 1:** Demographic data

Characteristics	Number of patient (%) ( *n* = 105)
*Age (y)* :	
Mean	45.5
Median	45
Minimum–maximum	17.0–92.0
*Gender* :	
Female	34 (32.4)
Male	71 (67.6)
*Injury severity score* :	
Mean	21.7
Median	22
Minimum–maximum	1–75
Standard deviation	12.7
*Final computed tomography result* :	
Improved	11 (10.5)
Resolved	73 (69.5)
Stable	21 (20.0)

**Fig. 2:**
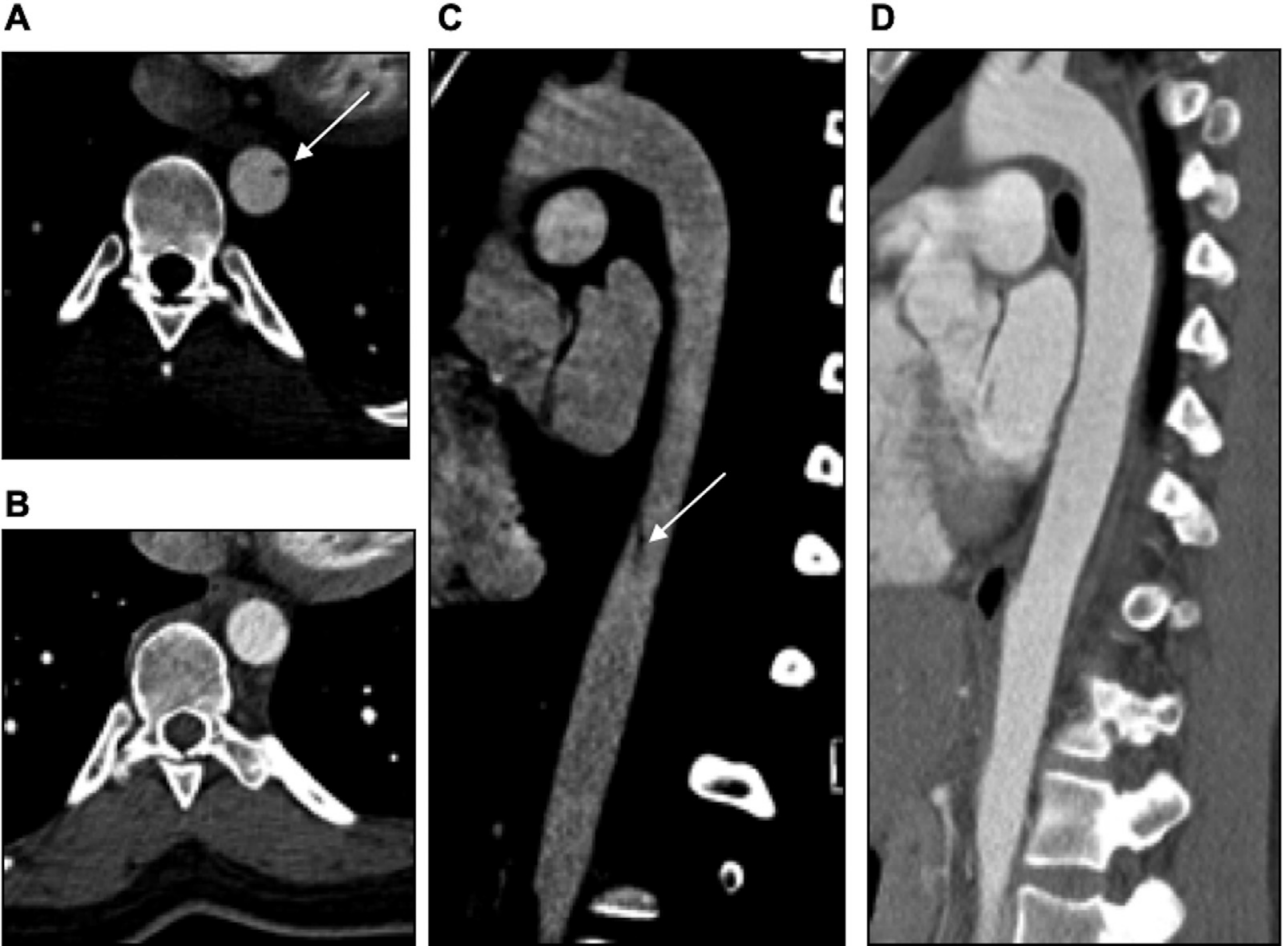
Grade I minimal aortic injury in the descending thoracic aorta on baseline trauma CTA in axial ( **A** ) and parasagittal oblique planes ( **C** ) (white arrows). Forty eight-hour follow-up computed tomography angiography demonstrated complete resolution of the MAI ( **B, D** ).

**Fig. 3:**
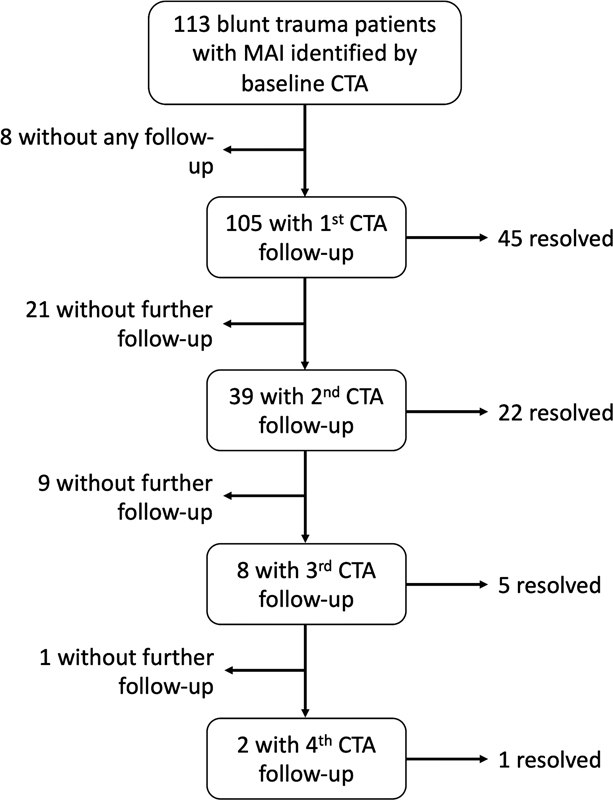
Flowchart summarizing follow-up computed tomography angiography (CTA) of the studied trauma patients.

**Fig. 4:**
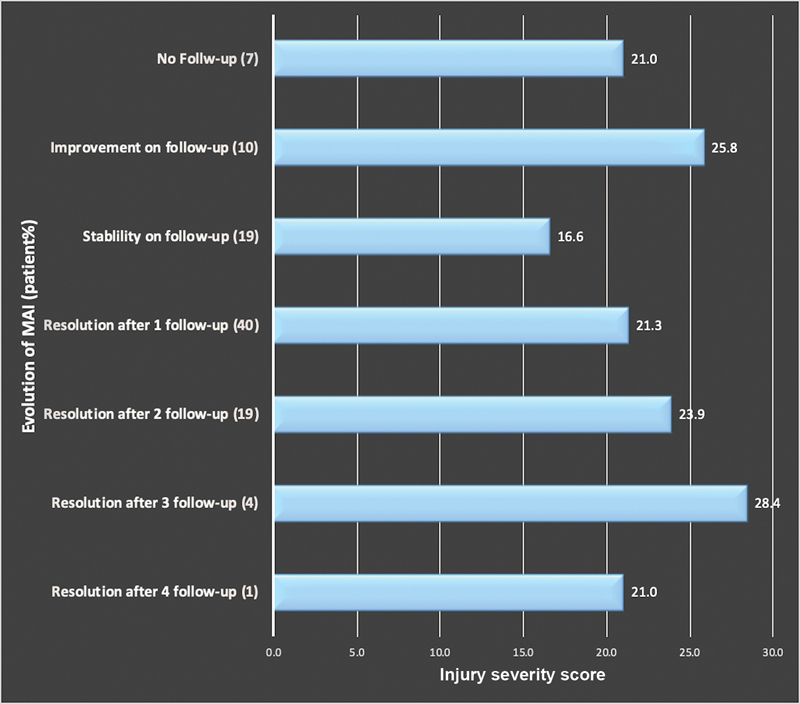
Average injury severity score (ISS) for surviving trauma patients. While there appears to be a trend regarding time to resolution of minimal aortic injury (MAI) and ISS, it was not found to be statistically significant ( *p* =0.43).

Among the 105 surviving MAI trauma patients who received follow-up chest CTA imaging, 45 patients (42.9%) showed resolution or complete healing of the MAI on their first follow-up chest CTA ( Fig. 2A, D ), with an average ISS of 21.3. A second follow-up CTA was performed on 39 patients (unresolved MAI), and of these, 22 had resolution of their MAI, with an average ISS of 23.9. A third follow-up CTA chest was obtained on eight remaining patients (unresolved MAI), and five demonstrated resolution, with an average ISS of 28.4. Finally, two patients (unresolved MAI) had a fourth follow-up CTA, and one showed resolution of MAI at this time, with an ISS of 21 ( Table 2 ; Fig. 3 ). The first, second, third, and fourth follow-up CTAs were performed at a median of 2, 10, 28, and 261 days, respectively ( Table 3 ) and none of the patients in this cohort of 105 patients succumbed to their aortic injury over the period of study. Three patients expired after discharge from other causes. Furthermore, at the time of the first follow-up CTA, which was performed on a total of 105 patients, 42 (40%) demonstrated stability (average ISS 21.3), and 18 (17.1%) had improvement (average ISS: 23.7; Table 2 ). No follow-up imaging was obtained on eight patients (average ISS: 21), who comprised 7% of the 113 patients identified as having MAI at the baseline scan ( Fig. 4 ). More importantly, no MAI patient demonstrated progression of the initial MAI to a higher-grade aortic injury on follow-up chest CTA imaging. We define the effective follow-up number as the number of follow-ups by which point the patient has no further change in their condition. By this metric, 94.3% of patients showed no further changes after their second follow-up CTA ( Table 4 ).

**Table 2:** Computed tomography angiography results for each follow-up

CT result	Number of patients	Percentage	ISS score	
			Mean	SD
*First CT follow-up* :				
1 (resolved)	45	42.9	21.29	11.42
2 (improved)	18	17.1	23.72	17.13
3 (stable)	42	40	21.26	12.04
Total	105		21.70	12.69
*Second CT follow-up* :				
1 (resolved)	22	56.4	23.86	11.32
2 (improved)	5	12.8	33.40	9.86
3 (stable)	12	30.8	20.08	9.55
Total	39		23.92	11.13
*Third CT follow-up* :				
1 (resolved)	5	62.5	28.40	8.79
2 (improved)	1	12.5	21.00	–
3 (stable)	2	25	20.00	2.83
Total	8		25.38	7.93
*Fourth CT follow-up* :				
1 (resolved)	1	50	21.00	–
3 (stable)	1	50	18.00	–
Total	2		19.50	2.12

Abbreviations: ISS, injury severity score; SD, standard deviation.

**Table 3:** Duration between initial computed tomography angiography (CTA) and follow-up CTAs

Duration (d)	Number of patients	Mean	Median	Minimum	Maximum
Follow-up 1st	105	2.1	2.0	0.0	17.0
Follow-up 2nd	39	27.8	10.0	1.0	219.0
Follow-up 3rd	8	50.4	28.0	8.0	158.0
Follow-up 4th	2	261.5	261.5	223.0	300.0

**Table 4:** Number of follow-up computed angiography scans needed to reach the point of “no progression”

^a^ Effective number follow-up	Number of patients	Percentage	Cumulative frequency	Cumulative percent
1	74	70.48	74	70.48
2	25	23.81	99	94.29
3	5	4.76	104	99.05
4	1	0.95	105	100
*Analysis variable: effective number follow-up* :				
Mean	Median	Minimum	Maximum	Standard deviation
1.3619048	1	1	4	0.6220903

All 105 patients with follow-up CTA results and ISS scores were included in the logistic analysis to determine a relationship between their condition at most recent assessment and ISS score, with the former as the outcome and the latter as the explanatory variable. The results indicate that there is no statistically significant relationship ( *p* = 0.2231). An ordinal logistic regression was performed on the 39 patients who had a second follow-up CTA, assigning the second follow-up CTA result as the outcome, and the first follow-up CT result as the predictor. This analysis showed no significant relationship between the first and second follow-up CTA results ( *p* -value = 0.7851). We also investigated age, gender, and ISS score as potential explanatory factors for the most recent CTA result available. Of the three factors, only age was found to be significant ( *p* -value = 0.0022), with a corresponding odds ratio of 0.96. This indicates that the odds of MAI resolution decrease by approximately 4% for each year of advancing patient age.

## Discussion

**Fig. 5:**
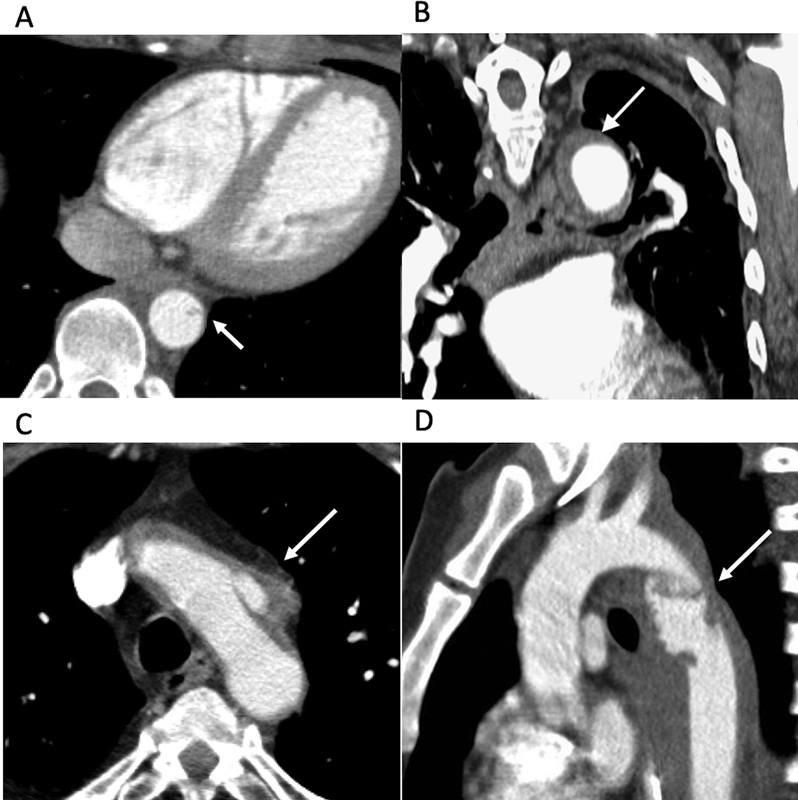
Minimal aortic injury characterized by a <1.0 cm intimal flap in the descending thoracic aorta (white arrow). ( **A** ) No external contour changes. ( **B** ) Intramural hematoma in the proximal descending thoracic aorta, consistent with a Type II injury (white arrow). ( **C** ) It demonstrates a posttraumatic pseudoaneurysm, or Type III injury. There is an obvious, focal contour abnormality in the aortic arch, although no free aortic rupture (white arrow). ( **D** ) It reveals a Type IV injury—consistent with free aortic rupture. Note the extensive contour abnormality in the proximal descending aorta and large peri-aortic hematoma (white arrow).

**Fig. 6:**
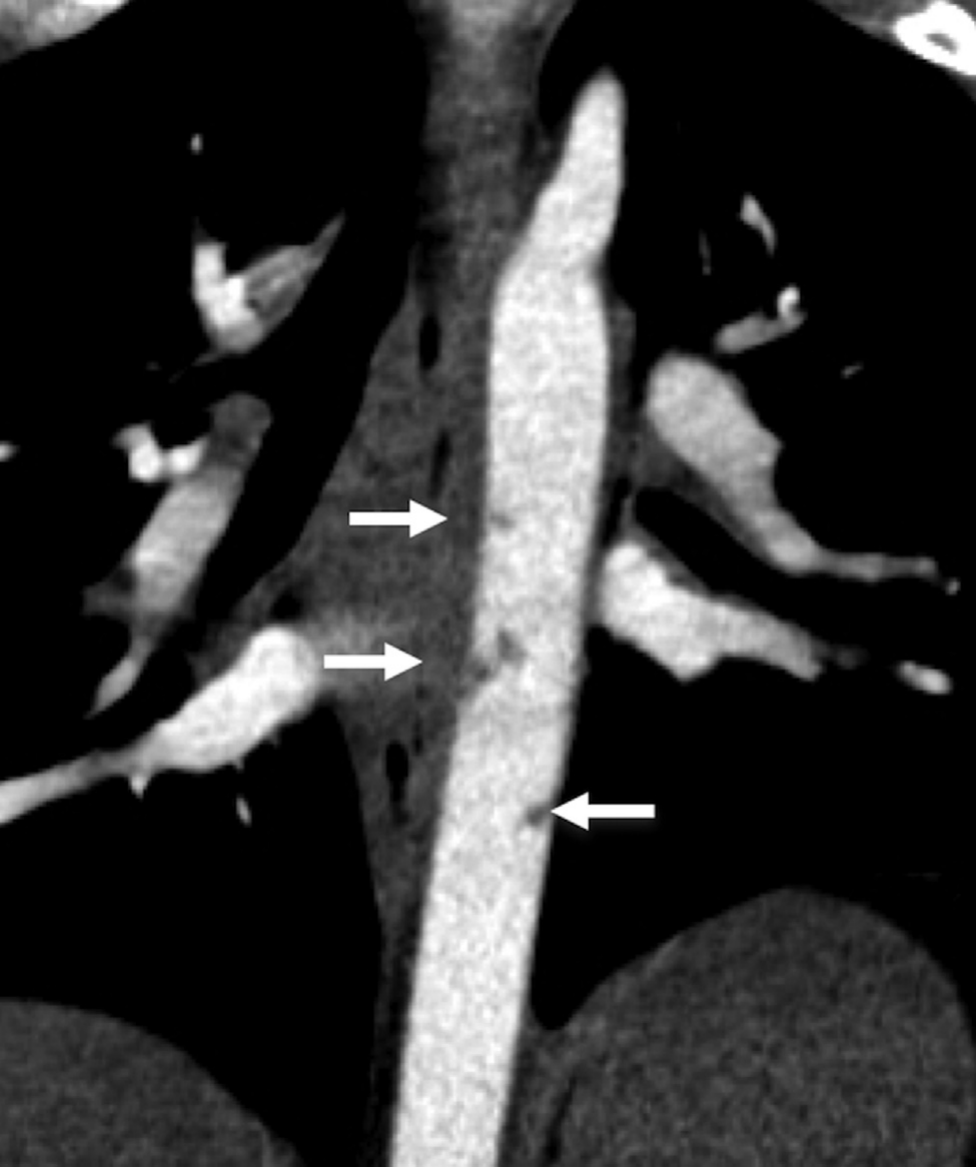
Coronal multiplanar reconstruction computed tomography angiography demonstrates multifocal minimal aortic injury in a trauma patient (white arrows). All injuries resolved on follow-up CTA imaging in 30 days.

The first description of acute traumatic thoracic aortic injury is believed to be by the ancient Egyptians in the 17th century BC, as chronicled within the Edwin Smith Surgical Papyrus. These chronicles describe traumatic injury to various parts of the body, including the aorta, as well as a knowledge of the vascular system and the concept of a pulse. 15 In 1958, Parmley et al 16 called attention to the importance of timely diagnosis of non-immediately fatal acute thoracic aortic injury caused by blunt trauma. Malhotra et al 3 subsequently described how evolution in diagnostic and operative techniques further changed the clinical management of ATAI up until 2001. Analysis of the National Trauma Data Bank reveals the overall incidence of acute or BTAI is 0.3% of all trauma admissions in the United States. 17 Despite the relatively low incidence, it cannot be over-emphasized that unrecognized or undiagnosed ATAI is an otherwise lethal injury, responsible for approximately 57% of all deaths at the accident scene. 18 Additionally, Burkhart et al 18 reported a 37% mortality in the first 4 hours for those trauma victims admitted to the hospital with aortic injuries. The high mortality and morbidity of acute injuries highlight the importance of prompt diagnosis and treatment. Appropriate antihypertensive therapy has also been shown to reduce imminent rupture, thereby saving lives. 7 19 Four grades of ATAIs have been described by Society for Vascular Surgery 20 ( Fig. 5A–D ). Grade I aortic injury is synonymous with MAI. MAI represents a subset of ATAI characterized by either: (1) localized intimal tears less than 1.0 cm or (2) intramural hematoma without external contour changes or associated periaortic hematoma ( Fig. 5A ). 1 21 On imaging, it is not uncommon to find multifocal MAI or a combination of MAI and higher degree of ATAI ( Fig. 6 ). 22

**Fig. 7:**
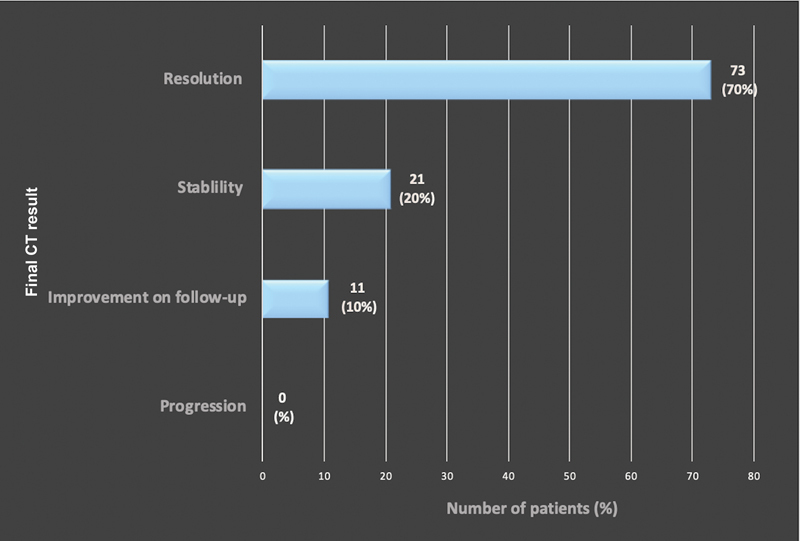
Evolution of minimal aortic injury amongst surviving trauma patients with follow-up computed tomography angiography (CTA) imaging. Seventy-three patients (70%) demonstrated resolution of minimal aortic injury (MAI) on follow-up imaging. Twenty-one patients (20%) demonstrated stability of MAI on follow-up imaging. Eleven patients (10%) demonstrated improvement on follow-up imaging.

Although often clinically occult, MAI is a distinct form of ATAI. As of this writing, limited case series have shown more favorable clinical outcomes for those patients with grade I MAIs compared with more classic grade II and III aortic injuries. Gunn et al 2 showed MAIs comprised 28.4% of all ATAIs in their patient population. These same researchers further observed no deaths related to MAI in the absence of intervention (endovascular repair or surgical repair). Similarly, Starnes et al 20 found most MAIs heal within 4 weeks after the initial injury. 20 Several additional case series also did not show progression of MAI in their respective patient populations. 2 23 24 25 26 27 Interestingly, this has also been supported by some animal models showing healing of MAI 19 days following the initial traumatic insult, promising rapid recovery. 28 Our experience mirrors conclusions reported elsewhere in the literature regarding the imaging characteristics and short-term natural history of MAI. Of 105 cases of traumatic MAI with follow-up CTA in our series over a course of 7 years, none demonstrated evidence of MAI progression to higher grades of aortic injury requiring more aggressive management or intervention. Most MAI victims (60%) showed either healing (42.9%) or improvement (17.1%) of the MAI on the initial follow-up chest CTA imaging in a median of 2 days. 26.6% of our blunt trauma victims demonstrated resolution of the MAI on further follow-up CTA imaging at 7 or 30 days. Twenty percent of our patients demonstrated a stable persistent MAI which neither progressed nor resolved on follow-up imaging ( Fig. 7 ).

Given the more favorable outcome of MAI, at our level 1 trauma center and in the literature, these injuries are most often managed conservatively. Specifically, management comprises blood pressure control and short-term follow-up sequential CTA imaging as needed. 3 7 29 Intravenous β-blockers (Esmolol in particular), due to their short half-life and rapid onset, are the most preferred medications. Esmolol is given as intravenous infusion at a rate of 25 to 50 µg/kg per minute and can be titrated up to 300 µg/kg per minute to keep systolic blood pressure under 100 mm Hg and heart rate under 100 beats per minute. 30 Diltiazem, nitroglycerin, and nitroprusside can also be used in conjunction with or as an alternative to IV β-blockers. 19 There is no consensus on the duration of antihypertensive therapy as of now, but aortic wall healing on imaging may be considered as an indicator for discontinuation. 31

Although no patient within our cohort showed progression of the initial MAI to a higher-grade injury, there have been some studies demonstrating progression. 2 3 32 33 34 Progression to higher grade injuries was reported in three of six patients (MAI to small pseudoaneurysm) by Malhotra et al, 3 one of seven patients (MAI to small saccular pseudoaneurysm) by Mosquera et al, 33 and two of fifty patients (grade I to grade II and grade III) by Osgood et al 34 on follow-up CT scans performed 16 days to 1 year later. Most of these imaging studies were conducted on older generation helical CT scanners, while other modalities such as transesophageal echosonography, transcatheter aortography, and intravascular ultrasonography were used for confirmation. All patients in these studies were treated conservatively without documented clinically significant morbidity or mortality during their follow-up. Our study showed 94.3% of patients reached some form of favorable outcome after their second follow-up CTA. Given the results of our study and previously published results, a need to define the utility of further follow-up CTA imaging in conservative management of MAI with injury progression needs to be addressed. Our data also reveals that odds of MAI resolution decrease by advancing patient age, suggesting that less intense follow-up imaging may be required in younger patients.

Despite a possible trend between increasing ISS and time to resolution of MAI, there was no statistically significant relationship between the most recent MAI assessment score and the ISS ( *p* = 0.2231). It is possible that our study is potentially underpowered with regards to detecting MAI progression. Perhaps, the inclusion of more blunt trauma victims with MAI may yield cases of progression which we did not encounter. A further powered study will help define an imaging-based clinical nomogram/algorithm to direct the management of those cases requiring continued follow-up imaging.

One additional limitation of our study is that only a percentage of MAI patients received the second follow-up CTA even though their injury was not fully resolved. In other words, the end result of that group of patients remains unknown to us.

## Conclusion

MAI is a subset of ATAI which is readily identifiable on MDCT angiography. Given our study's findings and those previously documented in the literature, it may be reasonable to assume there is limited efficacy with sequential follow-up CTA imaging for MAI, and such imaging may not be necessary in clinically stable patients. Limited reimaging may be even more justifiable in younger patients and in those with less severe injuries. Additionally, a more conservative approach to the management of MAI is supported now by several studies, including our own. Radiologists involved in the diagnostic interpretation of trauma chest CTAs must know how to readily differentiate MAI from other subtypes of ATAI which may necessitate more aggressive management. 35
