# Supplementary material for: Minimal Aortic Injury Detected on Computed Tomography Angiography during Initial Trauma Imaging: Single Academic Level 1 Trauma Center Experience
Source: Aorta (Stamford). 2022 Dec 20;10(6):265–73. doi: 10.1055/s-0042-1757793 (PMC9767788; doi:10.1055/s-0042-1757793)
Supplement: Supplementary file 1 — Supplementary Material [file 10-1055-s-0042-1757793-s210061.pdf]

CT_result_2 (First Follow-up)	CT_result_3 (Second Follow-up)	CT_result_4 (Third Follow-up)	CT_result_5 (Forth Follow-up)	Number of subjects
				8
improved				9
improved	improved			1
improved	improved	resolved		1
improved	improved	resolved	resolved	1
improved	resolved			4
improved	stable			2
resolved				44
resolved	resolved			1
stable				12
stable	improved			1
stable	improved	resolved		1
stable	resolved			16
stable	resolved	resolved		2
stable	stable			5
stable	stable	improved	resolved	1
stable	stable	resolved		1
stable	stable	resolved	resolved	1
stable	stable	stable		1
stable	stable	stable	stable	1
105	39	8	2	113

*The red color records were not re-counted in the Total.

Supplementary Fig. S1 Data frequency for reviewer observation. CT, computed tomography.

 © 2022. The Author(s).
